# GENEasso: a curated resource of credible disease–gene associations across complex diseases from GWAS summary statistics

**DOI:** 10.1093/nar/gkaf1097

**Published:** 2025-10-30

**Authors:** Tao Jiang, Mengting Shao, Junjie Wang, Pengze Wu, Changhui Zhu, Ranran Tang, Chen Cao, Ning Gu

**Affiliations:** Jiangsu Key Laboratory for Biomedical Electromagnetic Precision Theranostics, School of Biomedical Engineering and Informatics, Nanjing Medical University, Nanjing, Jiangsu 211166, China; Jiangsu Key Laboratory for Biomedical Electromagnetic Precision Theranostics, School of Biomedical Engineering and Informatics, Nanjing Medical University, Nanjing, Jiangsu 211166, China; Department of Medical Informatics, School of Biomedical Engineering and Informatics, Nanjing Medical University, Nanjing, Jiangsu 211166, China; Jiangsu Key Laboratory for Biomedical Electromagnetic Precision Theranostics, School of Biomedical Engineering and Informatics, Nanjing Medical University, Nanjing, Jiangsu 211166, China; Jiangsu Key Laboratory for Biomedical Electromagnetic Precision Theranostics, School of Biomedical Engineering and Informatics, Nanjing Medical University, Nanjing, Jiangsu 211166, China; Nanjing Women and Children’s Healthcare Institute, Women’s Hospital of Nanjing Medical University, Nanjing Women and Children’s Healthcare Hospital, Nanjing, Jiangsu 210004, China; Jiangsu Key Laboratory for Biomedical Electromagnetic Precision Theranostics, School of Biomedical Engineering and Informatics, Nanjing Medical University, Nanjing, Jiangsu 211166, China; Jiangsu Key Laboratory for Biomedical Electromagnetic Precision Theranostics, School of Biomedical Engineering and Informatics, Nanjing Medical University, Nanjing, Jiangsu 211166, China; Department of Cardiology, Cardiovascular Disease Center, Jiangsu Key Laboratory for Cardiovascular Information and Health Engineering Medicine, Nanjing Drum Tower Hospital, Medical School, Nanjing University, Nanjing, Jiangsu 210093, China; Nanjing Key Laboratory for Cardiovascular Information and Health Engineering Medicine, Institute of Clinical Medicine, Nanjing Drum Tower Hospital, Medical School, Nanjing University, Nanjing, Jiangsu 210093, China

## Abstract

Gene-based association analysis has become a powerful strategy to improve the biological interpretability of genome-wide association studies (GWAS) by aggregating variant-level signals at the gene level. Although several transcriptome-wide association study (TWAS)-specific databases have been developed, TWAS represents only one class of gene-based association methods and relies primarily on expression-mediated effects, whereas other gene-based approaches also play critical roles in identifying disease-associated genes. To address this limitation, we present GENEasso, a comprehensive platform that integrates multiple gene-level statistical frameworks to enable robust exploration of disease–gene associations across complex diseases. GENEasso systematically applies seven representative methods to 8226 curated GWAS summary statistics, generating 716 122 high-confidence disease–gene associations. The platform supports cross-method consensus scoring, tissue-specific enrichment prioritization, and ancestry-stratified analyses across five populations. Results can be interactively explored through Manhattan plots and ontology-based navigation, with full transparency and unrestricted access to data. A web server module allows users to upload their own GWAS summary statistics, select gene-based methods, and benchmark their findings against the GENEasso reference database. Concordance across methods increases the credibility of associations, improving reproducibility and supporting user-defined gene prioritization workflows. GENEasso is freely available as an open-access resource at https://www.geneasso.net.

## Graphical Abstract

**Graphical Abstract:**
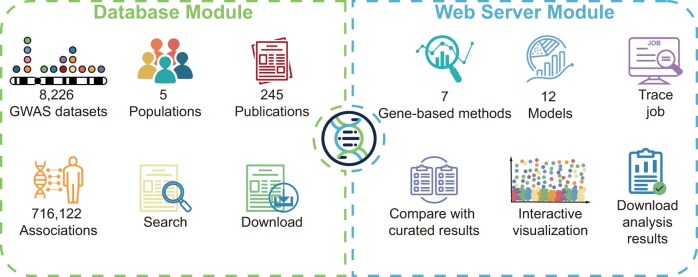

## Introduction

Genome-wide association studies (GWAS) have uncovered thousands of genetic variants associated with complex traits and diseases, providing an important resource for understanding human genetics [1]. GWAS databases such as the GWAS Catalog [2], GWAS Central [3], and GWAS Atlas [4] have become indispensable resources in this field. However, traditional single-nucleotide polymorphism (SNP) level GWAS results are often enriched for noncoding, intronic, or intergenic variants, which pose significant challenges for biological interpretation [5]. Moreover, GWAS evaluates each variant independently; yet, many complex diseases are driven by the combined effects of multiple SNPs, thereby limiting the power and biological relevance of single-variant analyses [6, 7].

To address this limitation, gene-based association methods have been developed to aggregate the effects of multiple SNPs at the gene level, thereby improving statistical power and interpretability. These methods are particularly valuable for complex traits, which are frequently influenced by numerous modest-effect variants acting in concert. Recent studies have demonstrated the utility of gene-based approaches in elucidating the genetic architecture of conditions such as Alzheimer’s disease [8–10], cardiovascular diseases [11–13], and cancers [14–16], among others [17–19].

Despite this progress, resources supporting gene-based association analyses remain limited in scope and diversity. In contrast to GWAS, which typically follow a standard analytic framework, gene-based association studies encompass a broad array of statistical models and genetic assumptions. Several platforms have been developed, such as TWAS-hub [20], webTWAS [21], TWAS Atlas [22], DisGeNET [23], Brain Catalog [24], and Genebass [25]—but each has limitations. TWAS-hub and webTWAS focus exclusively on transcriptome-wide association studies (TWAS), which represent only one category of gene-based association methods and rely on expression-mediated mechanisms. TWAS Atlas and DisGeNET are literature-curated resources, while Brain Catalog focuses exclusively on brain phenotypes. Genebass provides rare variant associations based on UK Biobank exome sequencing but lacks broader trait coverage. Importantly, these resources represent only a narrow slice of the gene-based methodological landscape.

To ensure a comprehensive representation of multiple gene-level genetic architectures, GENEasso integrates seven complementary gene-based association methods, carefully selected to reflect distinct statistical frameworks and genetic architectures. Four of them—MAGMA [26], PASCAL [27], SMR [28], and DEPICT [29]—are widely adopted and used in the field. MAGMA performs multi-marker regression accounting for linkage disequilibrium (LD), PASCAL aggregates association signals analytically to improve pathway-level power, SMR links GWAS and eQTL data to infer causal expression-mediated effects, and DEPICT prioritizes genes through co-regulation and pathway enrichment. These methods collectively capture different important aspects of gene-level association, from statistical signal aggregation to functional inference.

To improve the methodological diversity and biological relevance, GENEasso also incorporates three approaches: RWAS [30], which links regulatory elements such as enhancers and promoters to trait-associated variants; CWAS [31], which leverages chromatin state annotations to improve gene prioritization; and LDAK-GBAT [32], a flexible linear mixed model framework that supports alternative heritability assumptions for robust gene-level inference. By combining these seven methods within a unified framework, GENEasso enables multi-method gene-based analysis, facilitates cross-method validation, and improves the credibility and interpretability of gene–trait associations derived from GWAS summary statistics.

Despite the growing number of gene-based association methods, no centralized web platform currently allows researchers to apply and compare these tools in a convenient way. Most existing methods require complex software installation, custom input formats, and prior knowledge of reference panels, making limits accessibility for many researchers. Furthermore, the credibility and reproducibility of disease–gene associations critically depend on the ability to validate results across multiple methods, GWAS sources, and populations. To address these challenges, we developed GENEasso, a unified platform that integrates seven representative gene-based association methods (12 models) applied to 8226 curated GWAS summary statistics. The platform comprises two key components: a database module, which hosts 726 122 significant disease–gene associations, and a web server module, which allows users to upload their own GWAS summary statistics, apply multiple methods, and compare their results against those in the curated database. GENEasso supports comparisons across methods and GWAS summary statistics, tissue-specific enrichment analysis, and population-stratified association evaluations across five ancestries. These features allow researchers to assess the consistency of results across analytic strategies, data sources, and populations, thereby facilitating the identification of high-confidence candidate genes and improving the reproducibility of post-GWAS findings.

## Materials and methods

### Data processing

**Figure 1.**
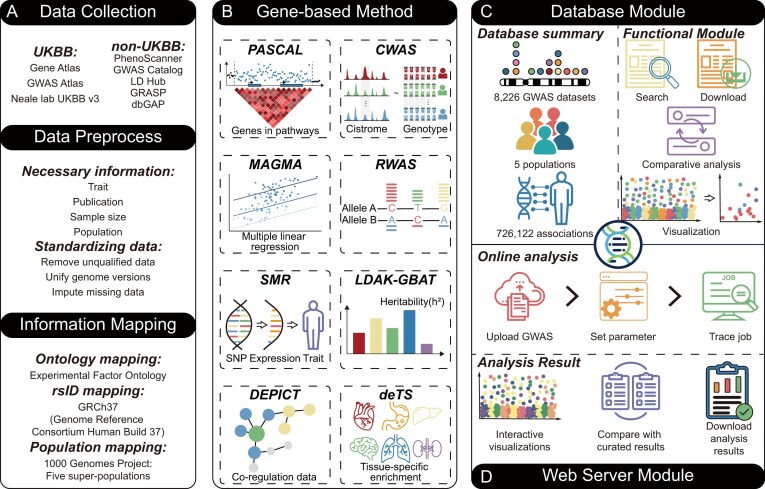
Overview of the GENEasso platform and analytical workflow. (**A**) GWAS summary statistics were collected from two major sources and then preprocessed to ensure data quality and standardized through trait, rsID, and population mapping. (**B**) Seven gene-based association methods, including PASCAL, MAGMA, SMR, DEPICT, CWAS, RWAS, and LDAK-GBAT, were applied to analyze the GWAS summary statistics. Tissue-specific enrichment analysis was performed using deTS. (**C**) The database module of GENEasso stores curated disease–gene associations and supports interactive exploration. (**D**) The web server module of GENEasso enables user-submitted analyses with customized pipelines.

To build the GENEasso database, we curated a total of 8226 GWAS summary statistics from publicly available sources, encompassing 14 complex human trait categories and spanning 245 original publications. Two sources of GWAS summary statistics were collected from UK Biobank (UKBB) cohorts and non-UK Biobank cohorts (Fig. 1A). UKBB-based GWAS summary statistics were collected from three commonly used resources: Neale Lab UKBB v3 (http://www.nealelab.is/uk-biobank/), Gene ATLAS [33], and GWAS ATLAS [4]. While all three derive from the same underlying UKBB cohort, they differ in sample inclusion criteria, association models, and quality control procedures, resulting in distinct statistical outputs. For non-UKBB datasets, we integrated GWAS summary statistics from multiple reputable public databases and consortia, including the GWAS Catalog [34], LD Hub [35], GRASP [36], PhenoScanner [37], and dbGaP [38], as well as individual project sources such as PGC (https://pgc.unc.edu), MAGIC (http://www.magicinvestigators.org), SSGAC (https://www.thessgac.org), and JENGER (http://jenger.riken.jp/en).

To ensure data quality and harmonization, we applied a multistep curation and standardization pipeline. For each GWAS dataset, we extracted key metadata—sample size, ancestry, trait definition, and publication source—from either the file header or the original publication. Datasets lacking clearly documented population information or sample sizes were excluded. When multiple datasets from different sources represented the same trait, including those derived from the UKBB cohort, we retained all available versions. Each dataset was curated and stored separately with source annotations to allow cross-study comparisons, ensure reproducibility, support confidence estimation of gene-level associations, and maintain robustness across resources.

Before gene-based analyses, all GWAS summary statistics were standardized with a single pipeline: variant coordinates were mapped to GRCh37 (GRCh38 converted via liftOver [39], non-bijective mappings removed) and rsIDs reconciled against dbSNP build 151, discarding records missing both coordinate and rsID. Alleles were harmonized to 1000 Genomes Phase 3 ancestry-matched references, requiring a reported effect allele; when only the effect allele was present, the noneffect allele was inferred. Strand-ambiguous palindromic SNPs with minor allele frequency (MAF) >0.40 were excluded. Reported allele frequencies were converted to MAF or imputed from 1000 Genomes when absent; variants were removed when the allele frequency reported in the study differed from the ancestry-matched 1000 Genomes reference by >0.20. For partially incomplete datasets, *Z*-scores were imputed where appropriate. Specifically, when *P*-values and effect sizes and directions were available, *Z*-scores were calculated using $Z = {\textrm {sign}}( \beta ) \times {{\Phi }^{ - 1}}( {1 - \frac{p}{2}} )$, where ${{\Phi }^{ - 1}}$ is the inverse standard normal cumulative distribution function, and $\beta $ denotes the effect size and $p$ denotes the *P*-value. Datasets lacking both $\beta $ and $p$ were excluded. Per-variant imputation quality reported by the sources (INFO/R^2^) was honored, and INFO <0.90 was applied for Neale Lab UK Biobank datasets. Sample-size fields (N or N_cases/N_controls) were retained for reporting. This imputation procedure was applied consistently across all datasets, ensuring harmonized and reproducible inputs for downstream gene-based analyses.

To improve trait interpretability and enable ontology-based querying, all reported traits from the original publications were manually mapped to the Experimental Factor Ontology (EFO) [40]. This standardization ensures the hierarchical organization of diseases and traits, reduces ambiguity, and facilitates the consistent integration of gene-based association results across studies.

Regarding population information, we mapped each GWAS summary statistic to one of the five super-populations defined by the 1000 Genomes Project [41] (AFR, AMR, EAS, EUR, SAS). We retained GWAS datasets from all available populations to support ancestry-specific analyses and enable cross-population comparisons of gene-level associations. Population assignments were based on metadata from the original studies and were standardized to ensure consistency across the database.

In addition to dataset-level filtering, we performed variant-level quality control (SNP-QC) on each GWAS summary statistic dataset. Specifically, we retained SNPs with a genotype calling confidence >0.9 and excluded those with MAF <0.01, Hardy–Weinberg equilibrium *P*-value <1 × 10^−7^, or duplicated identifiers, which were taken directly from the original publication or accompanying documentation. These filters ensured that downstream gene-based analyses were based on high-confidence variant-level inputs and minimized potential false-positive signals arising from low-quality data.

### Trait-specific tissue calculation

To identify biologically relevant tissues for each trait, we used PASCAL [27] and the deTS [42] algorithms, which perform tissue-specific enrichment analysis based on gene-level association results (Fig. 1B). Using the GTEx reference panel [43] (GTEx v8, 47 tissues), PASCAL first calculated trait-associated gene scores, selecting those with *P*-value <.05. Subsequently, we applied Fisher’s exact test to evaluate whether trait-associated genes were significantly enriched in specific tissues. For each trait, the top-enriched tissue was selected as its putative trait-specific tissue, which was then used in downstream tissue-dependent analyses, such as SMR and CWAS. In the web server, all tissues for a submitted GWAS are reported, and users may choose multiple tissues for downstream analyses; GENEasso executes each analysis per tissue and displays the results stratified by tissue.

### Database and web server architecture

GENEasso was implemented using a modular client–server architecture to support efficient data access and interactive analysis. The frontend was developed using the Vue.js framework, with user interface components styled via the Element UI library. The backend was built on the Spring Boot (Java) framework, enabling stable and scalable server-side operations. All curated GWAS summary statistics and gene-based association results are stored in a MySQL database, optimized for fast retrieval and filtering.

To handle user-submitted jobs, GENEasso employs an asynchronous task management system, allowing gene-based association analyses to be executed in the background without blocking the user interface. Each analysis task is assigned a unique job ID and tracked through a scheduling queue, with real-time progress and results accessible via the web interface. This design ensures responsive performance and supports multiple concurrent user analyses without compromising stability.

### Gene-based association method

To systematically identify disease-associated genes, GENEasso integrates seven gene-based association methods, each capturing different aspects of gene-level genetic architecture (Fig. 1B). These include MAGMA [26], PASCAL [27], SMR [28], DEPICT [29], RWAS [30], CWAS [31], and LDAK-GBAT [32]. All methods were applied using standardized pipelines and default parameters unless otherwise noted. Specifically, we implemented 12 models across seven methods—MAGMA, PASCAL, DEPICT, LDAK-GBAT, RWAS, CWAS (whole blood), and six SMR variants leveraging different eQTL resources (CAGE, Geuvadis, PsychENCODE) and GTEx-derived trait-relevant tissues (top-1/top-2/top-3 per trait).

MAGMA performs regression-based multi-marker gene analysis while accounting for LD; PASCAL aggregates SNP-level association signals into gene scores using analytic approximations based on chi-square statistics; SMR leverages Mendelian randomization to integrate GWAS with eQTL data, identifying genes whose expression may be associated with trait; DEPICT prioritizes genes through co-regulated expression profiles and evaluates tissue or cell type enrichment; RWAS and CWAS estimate gene–trait associations by modeling the relationship between genetic variants and regulatory features such as chromatin accessibility or epigenetic modification, followed by linking predicted regulatory activity to complex traits using GWAS summary statistics; and LDAK-GBAT estimates gene-level heritability contributions using a linear mixed model framework.

For tissue-aware methods such as SMR and CWAS, we used deTS to infer the top three enriched tissues per trait based on tissue-specific gene enrichment from GWAS results. Enriched reference panels were then selected accordingly to improve biological relevance. All computed gene-level associations were stored in the GENEasso database to facilitate multi-method, cross-trait, and cross-population comparisons (Fig. 1C).

### Web server configuration and parameter settings

The GENEasso web server supports six of the seven gene-based association methods implemented in the database: MAGMA, PASCAL, SMR, DEPICT, CWAS, and LDAK-GBAT. RWAS is not included due to its computational intensity, which is not suitable for real-time web-based analysis. Instead, FUSION-TWAS is provided as a supplementary option (Fig. 1D). Like RWAS, it integrates GWAS signals with transcriptomic features but with substantially greater computational efficiency. Whereas RWAS emphasizes chromatin accessibility and epigenomic regulation, FUSION-TWAS uses transcriptomic reference panels to impute gene expression and link it to complex traits.

To improve usability and reduce user burden, the web server is designed to require minimal parameter input. By default, all methods are executed using standardized settings, and the only required user-defined parameter is a significance threshold (cutoff), which defaults to 0.05 divided by the number of tested genes, corresponding to the Bonferroni correction. For specific methods, additional parameters can be adjusted as needed: SMR, CWAS, and FUSION-TWAS require users to select a relevant tissue, which can either be provided directly or inferred using the integrated tissue calculation module in GENEasso; MAGMA and LDAK-GBAT allow customization of the gene window size (default: 0 bp); and LDAK-GBAT also includes a power parameter for its model (default: 0.05). This design balances flexibility with ease of use, allowing users to perform reproducible and biologically meaningful gene-level association analyses without requiring detailed knowledge of method-specific configurations.

## Results

GENEasso is a comprehensive platform designed to facilitate gene-based association studies and exploration of complex disease–gene relationships. The resource integrates multiple statistical methods, curated GWAS summary statistics, and user-friendly visualization tools to support both large-scale database queries and customized online analyses.

The GENEasso resource is organized into seven primary modules—Home, Disease, Gene, Search, Downloads, Analysis, and Tutorial—providing intuitive navigation for users. The Analysis module is further subdivided into Analysis & Comparison, Trait-specific Tissue Calculation, and Job Search, enabling users to perform customized gene-based association analyses and enriched tissue calculation, and track job status.

### Database statistics

**Figure 2.**
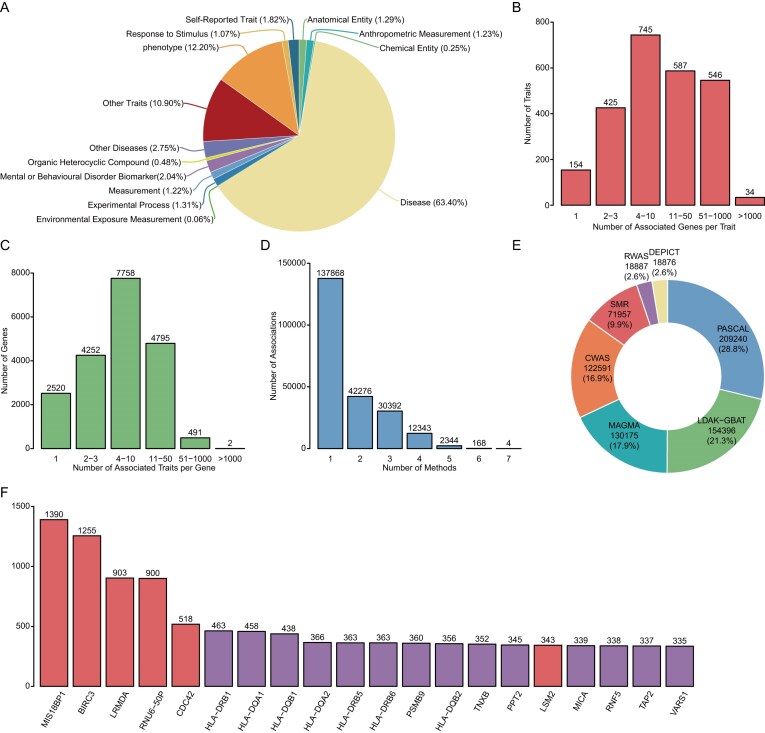
Statistical overview of the GENEasso platform. (**A**) Number of trait types. (**B**) Distribution of traits by number of associated genes per trait. (**C**) Distribution of genes by number of associated traits per gene. (**D**) Distribution of associations identified by different numbers of methods. (**E**) Number of disease–gene associations identified by each gene-based method. (**F**) Top 20 recurrent genes across traits (counted once per trait if identified by any method).

The current version of GENEasso contains 8226 curated GWAS summary statistics, encompassing 2491 unique EFO traits and 14 complex human trait categories (Fig. 2A). In addition, we classify the diseases category into 22 sub-disease categories (Supplementary Fig. S1). These datasets cover five populations from the 1000 Genomes Project (AFR, AMR, EAS, EUR, SAS), enabling population-stratified gene-based analyses. Among these summary statistics, 4294 belong to UKBB cohorts and 3932 belong to non-UKBB cohorts, with 95.34% of studies from the EUR super-population and 4.66% of studies from the other four human super-populations. Each GWAS summary statistic is annotated with metadata such as publication source, sample size, population, number of variants, and ontology-mapped trait descriptions.

Using seven gene-based association methods, PASCAL, MAGMA, SMR, DEPICT, CWAS, RWAS, and LDAK-GBAT, GENEasso has computed 726 122 significant disease–gene associations (Bonferroni-adjusted *P* <.05), 225 395 of which are unique pairings identified. On average, each disease trait is associated with 90.44 significant genes (Fig. 2B).

Among the top 20 recurrently associated genes, 6 genes reside outside the MHC (major histocompatibility complex) region. The predominance of MHC genes is expected given the region’s dense immune-regulatory variation and long-range LD, which collectively yield many correlated signals across immune-related traits [44]. BIRC3 regulates inflammation and cell-death signaling, and MIS18BP1 controls centromere licensing and chromosome segregation; these core processes confer broad biological pleiotropy and explain their frequent recurrence across diverse non-MHC loci (Fig. 2C and F) [45, 46]. Across the 22 categories, immune and inflammatory diseases are dominated by extended-MHC genes (HLA-DR/DQ cluster, PSMB9, TNXB, C4A, NOTCH4, TSBP1), indicating a pervasive immunogenetic architecture. Cancer and reproductive disease add prominent non-MHC signals at TERT and CLPTM1L alongside pan-category genes such as MIS18BP1 and BIRC3. Cardiovascular disease highlights CDKN2B-AS1, LPA, and SH2B3; respiratory system disease features CHRNA3; psychiatric disorders highlight APOE, APOC1. Digestive system disease, connective tissue disease, and integumentary system disease remain MHC-dense with accessory immune regulators (e.g. CFB), while recurrent non-MHC genes including MIS18B, BIRC3, LRMDA, and CDC42 appear across many categories, suggesting shared cellular processes beyond immune pathways.

We evaluated the overlap of significant trait–gene associations across the seven integrated gene-based methods. On average, 38.83% of associations were identified by at least two methods, and 20.08% were supported by at least three methods (Fig. 2D). Pairwise Jaccard index ranged from 0.01 (RWAS versus DEPICT) to 0.39 (MAGMA versus PASCAL), indicating generally modest overall overlap, with the highest similarity observed between MAGMA and PASCAL. These two methods co-identified an average of 30.93 genes per trait. In total, 14 859 high-confidence trait-gene associations were identified in four or more methods across traits (Fig. 2D and E). The moderate overlap highlights those different methods that often prioritize distinct signals due to their differing genetic architectures and model frameworks. The observed variability reinforces the necessity of a unified platform like GENEasso, which enables researchers to leverage the complementary strengths of multiple frameworks.

### Database user interface

**Figure 3.**
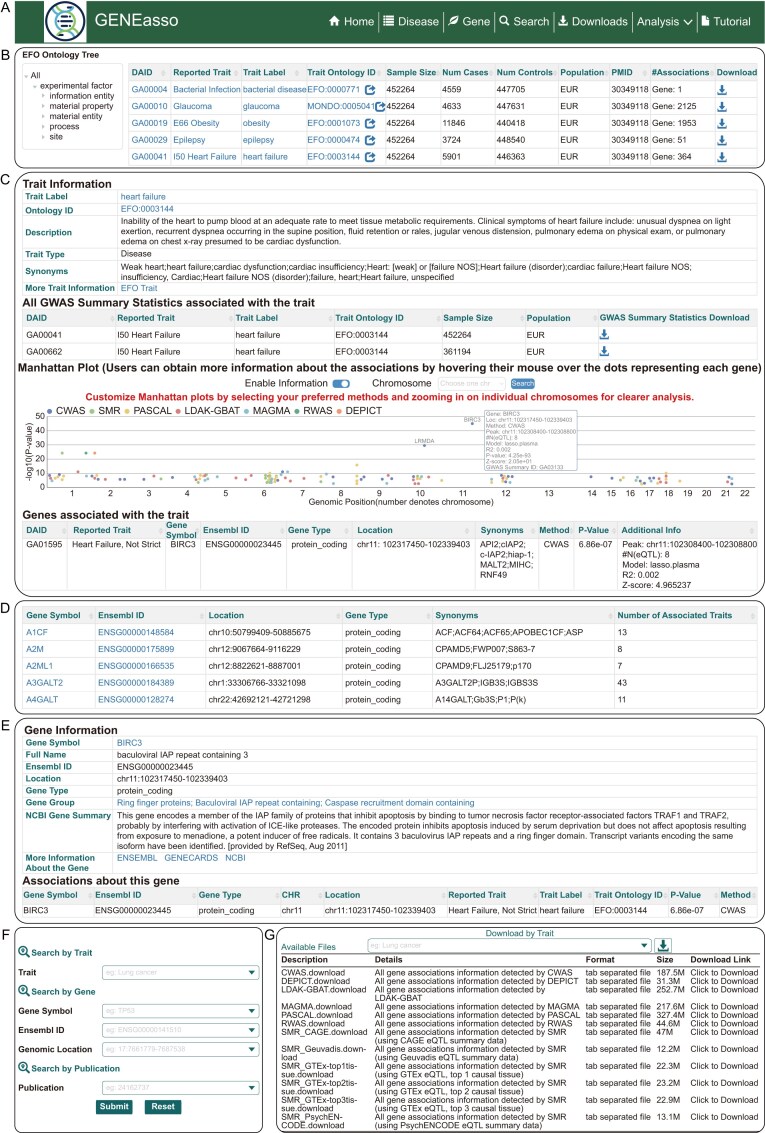
Main pages in GENEasso. (**A**) GENEasso navigation panel. (**B**) Overview of the Disease page. (**C**) Disease page example for the trait heart failure. (**D**) Overview of the Gene page. (**E**) Gene page example for the gene BIRC3. (**F**) Search page offering three query options: traits, genes, and publications. (**G**) Download page providing two categories of downloadable files.

GENEasso provides a friendly platform for exploring disease–gene associations from multiple perspectives, featuring disease-level and gene-level access through the Disease and Gene pages (Fig. 3A). On the Disease page, users can browse traits using the EFO ontology tree or the search box (Fig. 3B). Each dataset is annotated with a unique disease association ID, reported trait, standardized trait label, trait ontology ID, sample size, numbers of cases and controls, population, PubMed identifier (PMID), and number of gene associations. The GWAS summary statistics are available for download. Clicking a trait entry leads to a detailed view organized into four sections: trait information, all GWAS summary statistics associated with the trait, an interactive Manhattan plot for visualizing significant associations, and a sortable table listing associated genes across different methods with gene symbol, Ensembl ID, gene type, genomic location, synonyms, association method, *P*-value, and additional information (Fig. 3C).

The Gene page displays genes associated with at least one trait, with a sortable table that includes gene symbol, Ensembl ID, gene location, gene type, gene synonyms, and number of associated traits (Fig. 3D). Clicking on a gene leads to a detailed page displaying gene annotations, external database links (e.g. Ensembl [47], NCBI [48], and GeneCards [49]), and a table of disease–gene associations with *P*-values, trait label, trait ontology ID, and gene-based association methods (Fig. 3E). The Search page supports flexible querying across traits, gene symbol, Ensembl ID, gene location, and publications, with autocomplete suggestions to improve user experience (Fig. 3F). All tables are sortable, filterable, and fully downloadable (Fig. 3G). GENEasso also supports interactive visualizations that can be downloaded in high-resolution format. A unified global search bar enables users to locate relevant traits or genes efficiently across all modules.

In addition to trait- and gene-based browsing, GENEasso allows users to conveniently compare results across methods, studies, and populations for the same trait. For each disease entry, users can selectively display association results from different gene-based association methods, facilitating direct comparison of method-specific findings and identification of shared signals. This design helps users assess the reproducibility and robustness of associations. Furthermore, the database supports comparative analysis across multiple GWAS datasets, ancestries, or even distinct but related traits within a unified Manhattan plot, enabling users to evaluate the consistency and robustness of gene-level associations across studies and traits. For traits with a large number of associated genes, GENEasso provides a chromosome-level filtering option to simplify visualization and focus on the chromosome of interest.

### Web server module user interface

To complement the GENEasso database, we developed a robust and user-friendly web server enabling online gene-based association analysis and trait-specific tissue inference from user-submitted GWAS summary statistics. The web server comprises two main modules: Gene Association Analysis and Trait-specific Tissue Calculation.

Users begin by uploading GWAS summary statistics in plain text format. The computation process requires rsID, *P*-value, sample size, and beta fields; optional fields include effect allele, noneffect allele, and genomic coordinates. To facilitate ancestry-aware analysis, users specify population background (EUR, EAS, AFR, SAS, AMR, or Mixed), UKBB inclusion, and whether the dataset is sex-stratified. GENEasso implements automatic detection of column identifiers (e.g. rsID, *P*-value, beta, and CHR), minimizing user burden and reducing input errors. If ambiguous or unrecognized terms are encountered, the system issues a warning for user confirmation. For gene-based analysis, users could select one or more methods; results are visualized through an interactive Manhattan plot, with each method displayed in a distinct color. Hovering over each point reveals full association details, and results can be downloaded in Excel format.

The Trait-specific Tissue Calculation module uses the deTS algorithm, combined with the SMR and CWAS results in the database, to identify tissues likely to mediate trait-associated genetic signals. Upon submission of GWAS summary statistics, the system evaluates enrichment across 47 GTEx tissues. Results are presented in a table, where tissues with *P* <.05 are highlighted in red to emphasize statistically significant associations visually. This visualization facilitates intuitive prioritization of disease-relevant tissues, helping users to refine their interpretation and guide follow-up studies.

To facilitate efficient task tracking, GENEasso assigns a unique job ID upon submission and optionally notifies users via email. For each submission, the web server generates a per-method summary report capturing the selected identifiers (rsID, *P*-value, Beta, sample size; optional fields), the chosen method and parameters, and the significant genes with accompanying statistics. Results can be retrieved through the job ID on the “Job Search” page at any time. This design ensures users can flexibly monitor analysis progress and revisit results without maintaining an active session.

## Discussion

GENEasso addresses several challenges in gene-based association analysis by offering a unified framework that incorporates seven gene-based association statistical methods, each designed to capture different aspects of gene-level genetic architecture. By integrating these methods into the resource, GENEasso enables users to explore multiple analytical perspectives without requiring specialized coding expertise or complex software installations. Unlike TWAS-specific resources, GENEasso integrates statistical, regulatory, and chromatin activity approaches, provides ancestry-aware analyses across five populations, and supports ontology-based trait organization. Together, these unique features improve reproducibility and expand the utility of gene-based association studies. Notably, the platform allows gene prioritization in biologically relevant tissues by decoding trait-specific tissues based on input GWAS summary statistics, thereby increasing the interpretability and contextual relevance of the results.

Importantly, the significant genes calculated by GENEasso enables benchmarking and replication of novel analyses against a large, precomputed knowledge base. Researchers can cross-reference their candidate genes with associations identified across different methods, GWAS sources, and populations. The inclusion of population-specific results across five ancestries further improves the interpretability and relevance of findings, particularly in the context of ancestry-aware analyses. The ability to trace association signals across multiple dimensions, such as traits, methods, tissues, and populations, makes GENEasso a powerful resource for meta-analysis, cross-cohort comparison, and reproducibility assessments.

The web server module of GENEasso complements the database by enabling users to perform gene-based association analyses on their own GWAS summary statistics using the same standardized pipeline applied to curated public datasets. It supports multiple well-established methods, provides tissue-aware options where applicable, and allows selection of population-matched reference panels. Users can compare their results across methods, traits, and ancestries, and benchmark them against the resource-derived associations in the database. The platform is designed to be user-friendly, automating input recognition (e.g. rsIDs and gene aliases) and offering interactive visualization tools such as Manhattan plots. By lowering computational and technical barriers, the web server improves accessibility for researchers and facilitates reproducible, customizable gene prioritization in a wide range of study settings.

Despite its advantages, the GENEasso resource has several limitations. First, although the database includes ancestry-specific results, data coverage remains skewed toward European-derived GWAS, limiting the resolution of non-European disease–gene associations. Second, GENEasso focuses on common variant associations derived from GWAS summary statistics and does not yet incorporate results from rare variant, burden-based analyses, which are increasingly important for understanding disease biology. Lastly, while several tissue-aware methods are supported, tissue calculation accuracy is restricted by the availability and consistency of eQTL reference panels, particularly in non-European tissues.

We aim to improve the GENEasso database by incorporating additional high-quality GWAS datasets from non-European populations, extending support to rare variant-based methods, and exploring consensus-building strategies across models. We also plan to integrate additional molecular annotations, such as methylation QTLs, chromatin accessibility data, and single-cell tissue specificity, to refine the resolution and biological interpretability of gene-level associations.

In summary, the GENEasso database module provides a useful and flexible resource for exploring disease–gene associations using multiple methods and diverse GWAS datasets. Although it does not capture all possible association scenarios, it can assist researchers in conducting cross-method evaluations and identifying candidate genes with greater confidence.

## Supplementary Material

gkaf1097_Supplemental_Files

## Data Availability

The data underlying this article are available in GENEasso (https://www.geneasso.net) and can be freely downloaded. Scripts for running the gene-based association methods to generate disease–gene associations are provided on the Downloads page (https://www.geneasso.net/#/downloads). No registration or login is required.
