# Supplementary material for: GENEasso: a curated resource of credible disease–gene associations across complex diseases from GWAS summary statistics
Source: Nucleic Acids Res. 2025 Oct 30;54(D1):D1415–24. doi: 10.1093/nar/gkaf1097 (PMC12807607; doi:10.1093/nar/gkaf1097)
Supplement: gkaf1097_Supplemental_Files [file gkaf1097_supplemental_files.zip › Supplementary material.pdf]

Supplementary Material

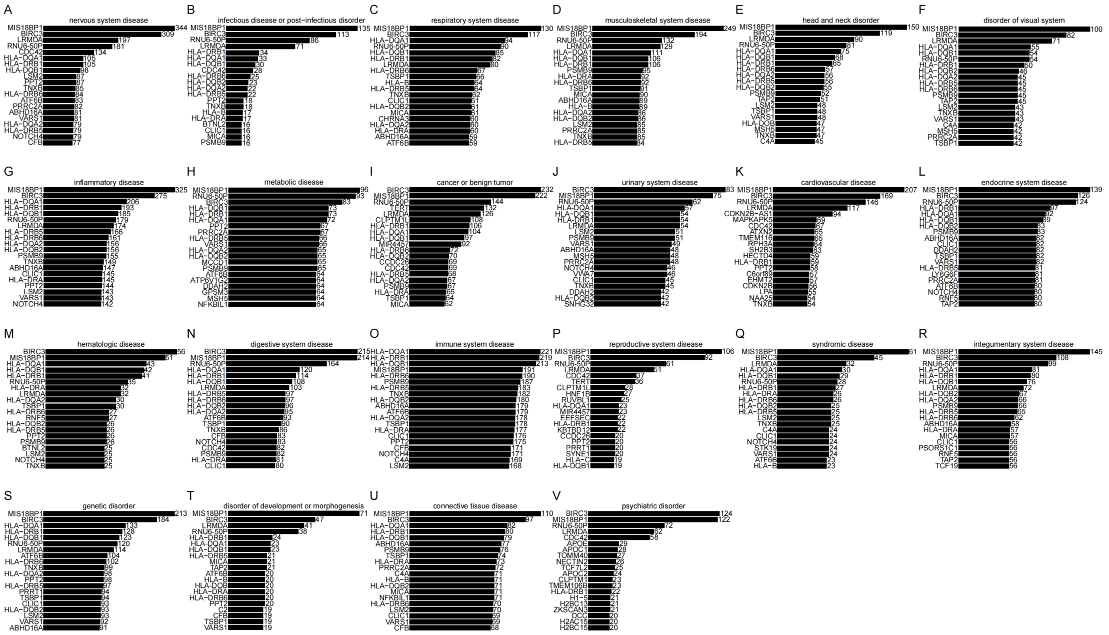

Figure S1. Top 20 recurrent genes across different disease. (A) nervous system disease. (B)infectious disease or post−infectious disorder. (C) respiratory system disease. (D) musculoskeletal system disease. (E) head and neck disorder. (F) disorder of visual system. (G) inflammatory disease. (H) metabolic disease. (I) cancer or benign tumor. (J) urinary system disease. (K) cardiovascular disease. (L) endocrine system disease. (M) hematologic disease. (N) digestive system disease. (O) immune system disease. (P) reproductive system disease. (Q) syndromic disease. (R) integumentary system disease. (S) genetic disorder. (T) disorder of development or morphogenesis. (U) connective tissue disease. (V) psychiatric disorder.
